# Overcoming the central planner approach – Bilevel optimization of the European energy transition

**DOI:** 10.1016/j.isci.2024.110168

**Published:** 2024-06-03

**Authors:** David Yang Shu, Christiane Reinert, Jacob Mannhardt, Ludger Leenders, Jannik Lüthje, Alexander Mitsos, André Bardow

**Affiliations:** 1 Energy & Process Systems Engineering, Department of Mechanical and Process Engineering, ETH Zurich, 8092 Zurich, Switzerland; 2 Institute for Technical Thermodynamics, RWTH Aachen University, 52062 Aachen, Germany; 3 JARA-CSD, 52056 Aachen, Germany; 4 Process Systems Engineering (AVT.SVT), RWTH Aachen University, 52074 Aachen, Germany; 5 Institute of Energy and Climate Research, Energy Systems Engineering (IEK-10), Forschungszentrum Jülich GmbH, 52425 Jülich, Germany

**Keywords:** Energy systems, Energy Modeling, Economics

## Abstract

The energy transition is a multinational challenge to mitigate climate change, with a joint reduction target for greenhouse gas emissions. Simultaneously, each country is interested in minimizing its own energy supply cost. Still, most energy system models neglect national interests when identifying cost-optimal transition pathways. We design the European energy system transition until 2050, considering competition between countries in a shared electricity and carbon market using bilevel optimization. We find that national objectives substantially impact the transition pathway: Compared to the model solved using the common centralized optimization, the overall installed capacity increases by just 3% when including national interests. However, the distribution of the installed capacity changes dramatically by more than 40% in most countries. Our results underline the risk of miscalculating the need for national capacity expansion when neglecting stakeholder representation in energy system models and demonstrate the need for cooperation for an efficient energy transition.

## Highlights

• We compare a competitive energy system to a central planner approach
• Competition in electricity and carbon markets is modeled via bilevel optimization
• Impact of competition on optimal transition pathways shown in a European case study
• Capacity in most countries varies by over ±40% compared to a centralized approach

## Graphical abstract

**Figure:**
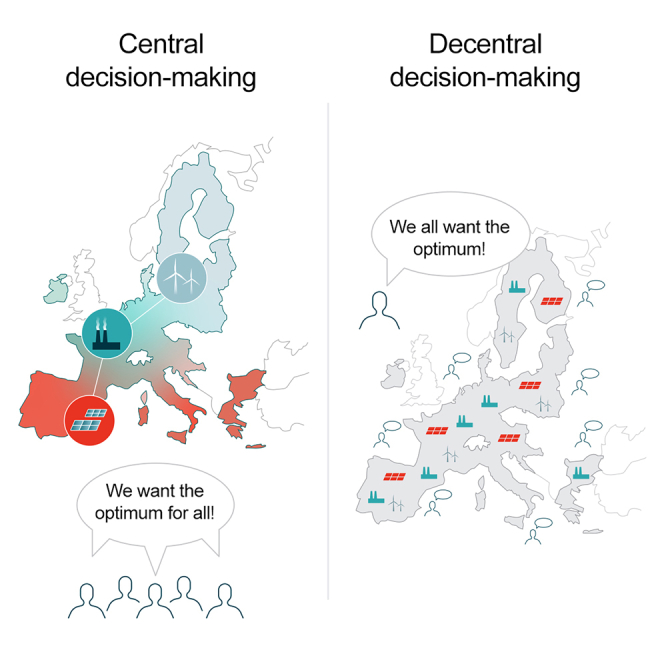

## Introduction

The European Green Deal^1^ calls for a major multinational transition of the European economy in all sectors – a transition that relies on the secure, clean, and affordable supply of energy. In parallel to the common goal of reducing Europe’s overall greenhouse gas (GHG) emissions in the interconnected electricity sector, all countries follow their own – partially competing – strategies to grant secure, clean, and affordable electricity to their electorate. The transition of the European energy system till 2050, therefore, needs to fulfill three requirements.

(1) meet the environmental goals set by policymakers,
(2) result in a cost-efficient system to maximize overall welfare, and
(3) account for the self-interest of sovereign countries to organize their own energy system.

To support policymakers in designing the energy transition,^2^ energy system models for long-term capacity expansion planning are essential. Still, in a review of energy system models and their contributions to answering policy questions,^2^ we find that only 5 of 40 reviewed energy system models explicitly address competition between actors. Hence, stakeholder behavior is commonly neglected, as most energy system models apply a central planner approach.

In reality, however, there is no central planner for the European energy system, and individual stakeholders act based on their own, sometimes conflicting, interests. Hofbauer et al. emphasize the importance of incorporating multiple governance scales into energy system models to provide meaningful decision support and facilitate coordination of actors across government scales.^3^ Nevertheless, multilevel governance and decision-making in the energy transition are often neglected in energy system models,^3^ resulting in unrealistic player behavior, an underestimation of overall costs,^4^ or failure to meet environmental targets.^5^

The European energy transition is an example of multilevel governance in energy systems: On the national level, the individual countries aim to ensure an affordable energy supply via capacity expansion and electricity trade. Meanwhile, at the European level, joint GHG emissions reduction targets are set while a market clearing mechanism minimizes the cost of the overall electricity supply. As a result, interests on the national and European levels can be misaligned, which cannot be accurately reflected by a central planner approach.

The misalignment of interests results in non-cooperative behavior that can be modeled by game theoretical approaches. Hierarchical relationships with a leader making decisions before a follower are called Stackelberg games^6^ that are considered a modeling approach in energy system models (e.g., ^7^^,^^8^^,^^9^). When the leader has complete knowledge of the followers’ behavior, the Stackelberg game can be formulated as bilevel optimization problems. In general, a bilevel optimization problem contains an upper-level problem describing the leader’s decisions and a lower-level problem describing the followers’ decisions.^10^ As such, bilevel optimization can model decision-making on two levels and support market-based capacity expansion planning by considering the objectives of individual countries and the common markets on different hierarchical levels. Bilevel optimization is an established method for electricity system and market analysis, with applications ranging from market bidding and electricity system expansion to the optimal expansion and operation of electricity storage for arbitrage.^11^^,^^12^^,^^13^ The formulation of bilevel optimization problems is flexible and depends on the analysis at hand. Multi-leader-single-follower structures are well suited to represent capacity expansion with participants interacting in a shared liberalized market. In such multi-leader-single-follower games, the investment decisions of individual players are modeled on the upper level, and the common electricity market is modeled on the lower level.^11^

Most applications of bilevel optimization to capacity expansion problems of electricity and energy systems (Table 1) investigate oligopolistic markets, i.e., markets dominated by few participants. The objective function of participants on the upper level is typically cost minimization, and the objective function of the market on the lower level is typically to maximize social welfare by minimizing operational costs.

**Table 1:** Literature overview putting the present study in context – Bilevel optimization in capacity expansion problems of electricity systems

source	focus/case study	players/time steps/technologies	game design	UL objective	UL variables	LL objective	LL variables
Kazempour et al.^7^	capacity expansion in an oligopolistic market	2/7/2	single-leader, single-follower	maximize the profit of the producer considering investment and operation under uncertain rival behavior	investment decisions and price offer	minimize social welfare costs in the electricity market	operation decisions of producers
He et al.^8^	capacity expansion under a carbon tax	3/3/2	single-leader, multi-follower	minimize collected carbon tax by policymaker	carbon tax rate	maximize the profit of the producer and transmissions company	investment and operation decisions of producers and transmission of electricity
Wogrin et al^14^; Wogrin^15^	capacity expansion in an oligopolistic market	2/4/2	multi-leader, multi-follower	maximize the profit of the producer considering investment & operation	investment decisions	maximize the profit of producers	operation decisions of producers
Wogrin et al.^16^	capacity expansion in an oligopolistic market	2/12/3	single-leader, single-follower	maximize the profit of players considering investment and operation	investment decisions	minimize social welfare cost	operation decision of the producer
Rocha et al.^17^	capacity expansion under a cap-and-trade program	4/2/3	multi-leader, multi-follower	maximize the profit by the producer considering investments, operation, and allowance trade	investment decisions	minimize social welfare costs in the electricity and carbon allowance market	operation decisions of producers
Panos et al.^4^	capacity expansion in an oligopolistic market	5/2/14	multi-leader, multi-follower	maximize the profit of countries considering investment and operation	investment decisions	maximize social welfare	operation decisions of producers and transmission of electricity
Taheri et al.^18^	Trilevel problem: Transmission (third level) and capacity expansion in an oligopolistic market (second level)a	2/3/2	single-/multi-leader, common follower	maximize the profit of the producer considering investment and operation	investment decisions and price offer	maximize social welfare	operation decisions of producers and consumers
Martelli et al^9^	capacity expansion under incentives for renewables	2/2/10	single-leader, single-follower	minimize the cost of incentives/maximize collected carbon tax by policymaker	incentive and carbon tax rates	minimize the cost for producers	investment and operation decisions of producers
this study	capacity expansion in a competitive market under GHG emission constraint	21/10/10	multi-leader, single-follower	minimize cost of stakeholders (investment, operation, congestion, and carbon allowances)	investment decisions	minimize social welfare cost	operation decision of producers

The most relevant features of each study are underlined. UL: upper level; LL: lower level.

While bilevel optimization is well suited to reflect multilevel governance, it entails a complex mathematical formulation and requires sophisticated solution methods tailored to the bilevel problem type. For example, some of the authors developed the first rigorous algorithms for nonlinear bilevel problems with a nonconvex lower level^19^ and mixed-integer nonlinear bilevel problems.^20^ In energy systems modeling using bilevel optimization, both upper- and lower-level problems are typically formulated as linear problems.^16^ Bilevel optimization problems with linear (or more generally convex) lower-level problems can be reformulated to an equivalent single-level nonlinear optimization problem and solved using commercial solvers. Still, the high computational effort has limited the size and complexity of bilevel optimization problems. For instance, of the studies reviewed in Table 1, only two consider energy storage despite the value of storage in energy systems with high penetration of renewables. The two studies including storage are limited to two players.^9^^,^^16^ Detailed models of electricity storage have been included in bilevel optimization problems,^12^^,^^13^ with a limited focus on the placement of the batteries or their bidding strategies to enable arbitrage. The present study neglects energy storage to reduce computational time, although introducing storage constraints in our model is straightforward. Overall, bilevel optimization of the capacity expansion in energy systems is typically limited to stylized systems,^7^^,^^14^^,^^16^^,^^18^^,^^21^ or small-scale case studies^4^^,^^17^ with no more than 5 players.^16^

Still, studies using bilevel optimization demonstrate the potential impacts of misaligned interests on the overall welfare. For example, Panos et al. observe a decrease in social welfare by 10% in a capacity expansion problem with a common electricity market. However, their study is limited to two timesteps, a single fixed carbon price, and five countries exerting market power.^4^ Bilevel optimization can also be used to model carbon emission regulations: He et al. compare a cap-and-trade system to a taxation system.^8^ For taxation, bilevel optimization is used to model the selection of tax rates on the upper level, while the generation companies’ investment and operation decisions and the grid owner’s operation decisions are determined on the lower level. Martelli et al. develop a bilevel formulation where the upper level sets the tax rate for a district energy system minimizing investment and operation costs.^9^ Rocha et al. model a cap-and-trade system with 4 players optimizing their capacity expansion under a cap-and-trade program in a 9-node network model of Northern Illinois, where players must choose between predefined capacity expansion plans.^17^

## Case study

This study assesses the impact of decentral planning and market representation in energy system models. In our assessment, we focus on electricity, a sector within the energy system, for simplicity. However, the methodology can be applied to a broader scope considering sector-coupled energy systems. We assess the impact by comparing the investment strategies derived from the decentralized and centralized modeling approach in a real-life case study. To ensure comparability between the results, we assume the same parameters in both models for availabilities, transmission capacities, prices of fossil fuel imports, and the investment and operating costs of power plants.

In particular, our case study assesses the impact of market representation in the European energy transition toward net-zero operational emissions by 2050. In the transition, we focus on pan-European decarbonization and thus neglect national decarbonization targets.

Importantly, the modeling approaches differ in their consideration of carbon allowance and congestion costs: The centralized model limits GHG emissions globally via a constraint on the total emissions from all countries, eliminating the need for a carbon price. Further, the exchange of electricity is governed by electricity balances, neglecting explicit electricity prices. Thus, the centralized model neglects explicit carbon costs and congestion costs. However, in the decentralized model, the individual countries follow their individual objectives on the upper level. Here, the global emission and energy balance constraints cannot be incorporated directly. Instead, the decentralized model introduces markets between countries on the lower level of the bilevel problem. On the lower level, GHG emission constraints are included to represent the carbon allowance market, resulting in an endogenous carbon allowance price. Further, electricity balances are added on the lower level, representing a competitive electricity market to determine the operation of power plants, resulting in endogenous electricity prices. Grid congestion between countries and their market zones results in differences between locational market clearing prices, allowing for arbitrage during electricity trading. Thus, grid congestion in the decentralized model leads to congestion costs from electricity imports and exports.

To establish comparability between the two models, we retrospectively evaluate the centralized model’s congestion and carbon allowance costs as follows: We optimize the operation of the decentralized model for the fixed optimal electricity system design from the centralized model, thus yielding the market-based congestion and carbon allowance costs for the design of the centralized model.

In this work, we consider transactions on the carbon allowance markets as costs in the objective function of the countries. However, note that in practice, the transactions resulting from trading on the carbon allowance markets are transfers between companies or from companies to the government.

**Figure 1:**
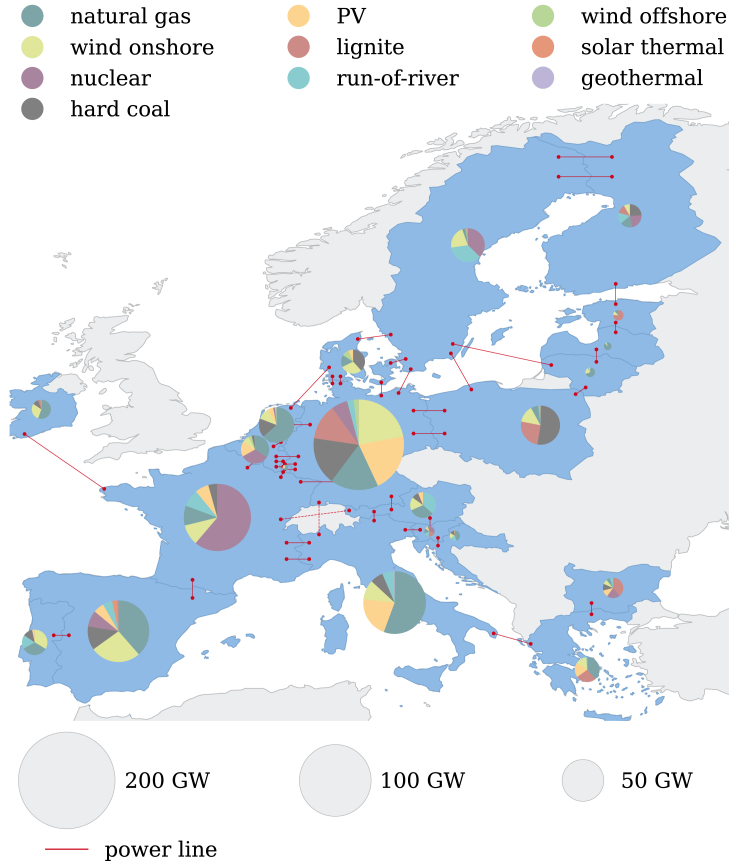
Existing electricity generation capacity for each country participating in the European electricity market in 2015

Our case study comprises 21 countries participating in the European electricity market. For simplicity, we assume that each country in the European electricity market corresponds to a single stakeholder since countries can specifically target different transition strategies through policies, and the non-regionally resolved bidding zones typically correspond to single countries. The countries aim to maximize their welfare by minimizing their individual total annualized cost. Each country is represented in the electricity system models with one node, including existing electricity generation technologies and capacity expansion limits. Electricity can be transmitted between the nodes via interconnections of power lines (Figure 1).

We model the European electricity system using a multi-leader-single-follower approach, where the 21 countries act as leaders in common electricity and carbon markets that act as a follower. With 21 players, 10 time steps, and 10 electricity generation technologies, our study surpasses typical proof-of-concept bilevel studies.

The interactions among countries with competing interests are modeled in a Nash game (see game-theoretical approach), for which we heuristically find an equilibrium solution using a Gauss–Seidel algorithm (see solution method). The interactions between each country and the electricity and carbon market are modeled as a Stackelberg game for which we formulate a bilevel optimization problem with linear upper- and lower-level problems. We reformulate the bilevel problem to a single-level mixed-integer linear programming problem to determine the investment strategies for the non-cooperative game.

We model operational GHG emissions according to the life cycle assessment methodology (ISO 14040^22^ and ISO 14044^23^) using the database ecoinvent 3.7.1 APOS^24^ and country-specific life cycle inventories for the electricity generation technologies based on Baumgärtner et al.^25^ As a life cycle inventory assessment method, we employ the Environmental Footprints 2.0 database.^26^ While this study focuses on climate change, our model can easily investigate additional impact categories.

We aggregate all annual time series with an hourly resolution to 10 typical time steps without temporal coupling. Further information on the implementation can be found in the method details.

## Results

In this section, we compare the European transition pathways resulting from the decentralized and centralized model. Furthermore, we consider the expansion strategies of individual countries and discuss the impact of non-cooperative behavior on electricity prices.

### Electricity system costs

**Figure 2:**
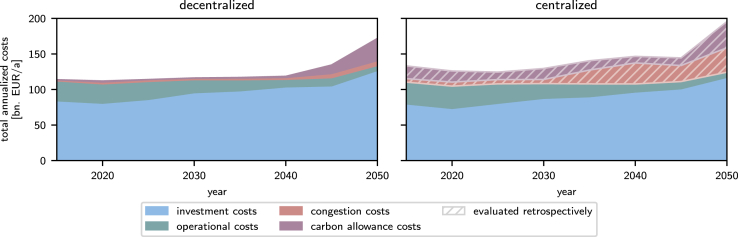
Total annualized cost of the European electricity system in the decentralized (left) and centralized (right) models The centralized model neglects the market models of the decentralized model, from which the congestion and carbon allowance costs emerge. To establish comparability, we retrospectively evaluate the centralized model’s congestion and carbon allowance costs (hatched): We optimize the operation of the fixed optimal decentralized model with the electricity system design from the centralized model.

The modeling approaches of the centralized and decentralized models differ fundamentally, in particular in consideration of the costs. For 2050, the investment and operational costs in the decentralized model are 8% greater than in the centralized model. The centralized model thus underestimates the investment and operating costs for systems that are, in reality, competitive (Figure 2).

In the centralized model, grid congestion costs occur indirectly due to constraints on transmission capacities (Equation 29), resulting in additional capacity expansion. In the decentralized model, congestion additionally results in costs due to arbitrage. Thus, the centralized model considers congestion but neglects markets and, therefore, arbitrage costs. For comparison with the decentralized model, we have determined the congestion costs retrospectively (Figure 2) by evaluating the infrastructure design of the centralized model in the decentralized model. Inserting the centralized model design as fixed in the decentralized model to determine carbon allowance and congestion costs for the centralized model retrospectively shows that overall costs, including congestion and carbon allowance costs, are higher for the centralized electricity system design compared to the decentralized design. In 2050, the total costs of the centralized electricity system design exceed the costs of the decentral design by 12%, as the decentral design directly considers market behavior. Centralized planning thus results in a design that incurs substantial additional costs when applied to a context with market behavior.

### European electricity system designs in 2050

Both the decentralized and the centralized electricity system designs rely heavily on onshore wind and photovoltaics for electricity generation. Further, no country relies on a single technology for electricity generation.

The decentralized electricity system design only has a 3% higher overall electricity generation capacity compared to the centralized design. This increase is negligible and is due to the inclusion of markets in the decentralized model, which encourages some countries to invest in additional renewable and fossil electricity generation technologies to mitigate high congestion costs. In contrast, the centralized model minimizes total annualized costs across all countries, neglecting congestion costs and reducing capacity expansion.

This impact of competition on the design and overall cost increases with time as the penetration of renewable electricity generation technologies rises due to more stringent emission limits. As a result, the transition pathways obtained from decentralized and centralized models increasingly diverge in later optimization years.

**Figure 3:**
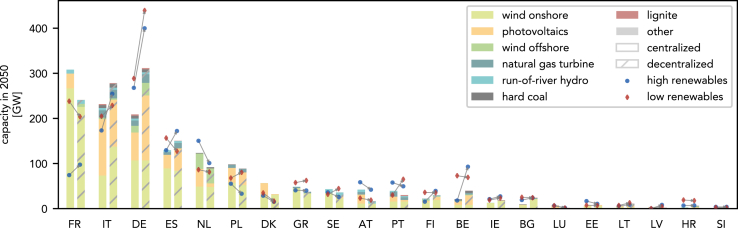
Capacities in the year 2050 in the decentralized and the centralized models and for two additional scenarios with high or low renewable availability Country abbreviations: France (FR), Italy (IT), Germany (DE), Spain (ES), Netherlands (NL), Poland (PL), Denmark (DK), Greece (GR), Sweden (SE), Austria (AT), Portugal (PT), Finland (FI), Belgium (BE), Ireland (IE), Bulgaria (BG), Luxembourg (LU), Estonia (EE), Lithuania (LT), Latvia (LV), Croatia (HR), Slovenia (SI)

We consider the mild increase of the overall capacity in the decentralized model to be negligible, in particular when considering modeling simplifications, such as the lack of storage or ramping constraints. This finding is also not surprising since the overall capacity is mostly determined by the demand, which is identical in both models. However, the results change substantially within individual countries: In 11 of the 21 countries, the installed capacity changes by over ±40% compared to the centralized electricity system design. Furthermore, the dominating electricity generation technology changes in 10 of the 21 countries (Figure 3). Thus, half of the countries end up with a completely different electricity system.

**Figure 4:**
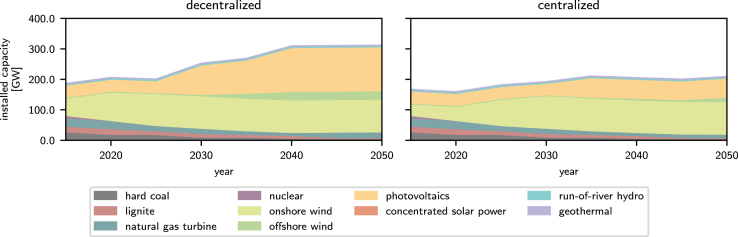
Capacity expansion of the German electricity system up to 2050 in the decentralized (left) and centralized (right) models

From the years with available data,^27^ we identified those with highest (1990) and lowest (2010) average capacity factors of PV, solar thermal, onshore and offshore wind power plants. Based on the identified years, we calculated the transition pathways for additional scenarios with high and low availability of renewable electricity generation. The capacity factors of renewable power plants substantially impact the outcomes of the energy transition, highlighting the need to consider uncertain parameters in the energy system design. However, regarding the focus of this study, the overall results are robust: Across all considered scenarios, competition greatly impacts the design within individual countries, with many trends persisting. Notably, capacity expansion in the three countries with the largest installed capacities follows the same trend, except for the high renewable scenario, where France relies on nuclear power instead of renewables, which leads to a significant reduction of the overall capacity by 61%–70% compared to the base scenario for the decentralized and centralized model, respectively. This significant capacity reduction is due to the higher full-load hours of nuclear power plants compared to wind and photovoltaic power plants.

### Electricity system design of individual countries: Germany

The cost of the decentralized model is 8% greater compared to the centralized model when setting aside congestion and carbon allowance costs. However, the differences between the decentralized and centralized models can be dramatic for individual countries, such as Germany. As Europe’s largest electricity consumer and interconnected with 9 neighboring countries in central Europe, Germany is discussed in detail in the following.

By 2050, the overall electricity generation capacity in the decentralized electricity system design exceeds the capacity in the centralized design by 49% in Germany (Figure 4). The difference is largely due to a greater capacity expansion of photovoltaics, resulting in other countries reducing their capacity expansion: The capacity of countries neighboring Germany is reduced in total by −13% in 2050, with Luxembourg (−76%), Austria (−60%), Denmark (−43%), and the Netherlands (−26%) seeing the largest reductions, primarily in photovoltaics. France, which has the largest total electricity generation capacity in the centralized electricity system design, sees a 20% reduction in the decentralized design, primarily in onshore wind and photovoltaic capacity. Thus, electricity generation capacity is moved to Germany despite the greater availability of renewables in neighboring countries: In France and the five countries with the largest relative reduction of installed capacity, the full load hours of photovoltaic power plants are 4%–31% greater than in Germany.

Increasing installed capacity in Germany in the decentralized design lowers congestion costs, as grid congestion and associated high electricity prices are reduced. In the decentralized model, reliance on countries with high availability of renewable electricity generation technologies results in high electricity and congestion costs from the perspective of the importing country due to high grid occupancy and arbitrage. Importing countries, such as Germany, are thus incentivized to expand domestic renewable capacities to reduce reliance on other countries in the decentralized model.

However, from a European perspective, capacity expansion in countries with low availability of renewable resources increases overall generation cost and is therefore avoided in the centralized model.

### Electricity prices

**Figure 5:**
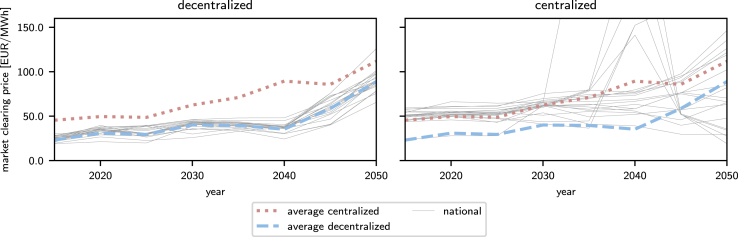
Average locational market clearing prices in decentralized (left) and centralized (right) models over the transition path The blue dashed and red dotted lines indicate the average market-clearing price for all countries in the decentralized and centralized models, respectively. Gray solid lines indicate the average locational market clearing prices in individual countries.

Fixing the centralized model design in the decentralized model to determine the locational market clearing prices for each country in the centralized model, we find that the average prices increase over time in both centralized and decentralized models (Figure 5). However, the average locational market clearing price is 40% lower in the decentralized model compared to the centralized model.

The locational market clearing prices differ between countries due to limited transmission capacities. The spread of prices in individual countries is much lower in the decentralized model, where the standard deviation of the prices increases from 3.1 EUR/MWh to 14 EUR/MWh from 2015 to 2050 (Figure 5). The spread is lower in the decentralized model, where individual countries expand their capacity, minimizing congestion costs as part of their objective function. High electricity prices thus incentivize additional capacity expansion. In the centralized model, the standard deviation increases from 7.7 EUR/MWh to 56 EUR/MWh in the same period and is 4 times higher in 2050. Here, the objective function neglects electricity prices during design optimization (see case study).

Thus, in the centralized model, the retrospective evaluated electricity prices reach extremely high average values in a few countries in 2035–2045, exceeding 150 EUR/MWh (Figure 5). The prices can be attributed to the lower capacity expansion in the centralized design, resulting in more frequent grid congestion.

In conclusion, the decentralized model results in lower locational market clearing prices with a lower spread, in particular as the share of renewables increases with tighter emission limits toward the end of the transition horizon.

Similarly, the carbon allowance price of the decentralized model is almost 16 times lower than the carbon allowance price evaluated retrospectively for the centralized model in the period from 2015 to 2040. However, in 2045–2050, the carbon allowance prices converge in both models to the penalty price for exceeding the GHG emission limit, as the electricity system is unable to compensate residual emissions (see method details).

## Discussion

The present publication introduces a model formulation for capturing the effects of decentral decision-making and market behavior in energy system models and further introduces a solution approach for the problem.

While the solution approach cannot guarantee convergence to a Nash equilibrium, the approach converges reliably in practice when applied to our problem. Furthermore, the solution to the decentralized optimization problem may not be unique: Several designs of the European electricity system may lead to a Nash equilibrium, which is a common issue in similar problems.^11^^,^^28^ In our case, we observe no substantial changes in the solution obtained when altering the order of players in the fixed-point iteration.

Our study highlights the importance of representing market behavior in electricity and by extension energy system analysis. However, we still simplify the real-world electricity market design. While major strategic decisions for energy systems are made at the country scale, the actual actors in the market are not countries themselves. Future studies should investigate the effects of competing players within countries on transition pathways. Adding industry stakeholders into energy system models would increase complexity but could result in further market effects that a centralized model cannot capture.

We assume that the trends we have identified in our work will strengthen significantly if the electrification of further sectors through sector coupling is considered, as the trading volume for electricity will then increase.

### Limitations of the study

The use of bilevel optimization in our modeling formulation to capture market behavior substantially increases model complexity. As a consequence, we simplify other aspects of the energy system model to maintain computational tractability. In our case study, simplifications are made consistently in both the centralized and decentralized models to maintain comparability of results:

In this publication, the energy system is limited to the electricity sector, although the modeling approach conceptually allows to model sector-coupling. Furthermore, time coupling is neglected to reduce problem size, prohibiting electricity storage. Additionally, we resolve the system at the country level, meaning that potential limitations of electricity transmission within the countries are neglected. However, the optimal design of renewable energy systems is impacted by the degree of modeling detail, e.g., by the spatiotemporal resolution, the availability of time-coupling, sector-coupling, and technical details, such as ramp constraints or start-up costs. Furthermore, energy system designs are sensitive to technical input parameters, such as discount rates, renewable technology availabilities and potentials, or technology costs.^29^^,^^30^^,^^31^ Our study considers only a few deterministic scenarios, neglecting the stochastic nature of long-term energy system planning. Therefore, while our study demonstrates the impact of market effects and misaligned interests on system design, future work should consider the sensitivity of the system design to the degree of modeling detail and the technical input parameters to supply insights to real-world systems.

Partial equilibrium models of the global energy sector exist that combine detailed bottom-up modeling with market representation, e.g., the TIMES^32^ model that can identify multiregional global equilibria. In particular, TIMES combines the central planner paradigm through a total net surplus maximization with a market representation that considers the price elasticity of demands and trade of energy commodities between regions. The single-level formulation enables higher modeling detail, including a market representation, but holds on to the central planner assumption.

In summary, models need to be tailored to the specific research question being studied, considering the trade-off between modeling stakeholder representation and other aspects of the energy system.

## Summary

During capacity expansion planning of energy systems, current models often neglect competing interests that arise from decentralized decision-making. To address this issue, we propose a bilevel formulation of the capacity expansion problem that considers competing interests in markets. We further present a solution algorithm to determine a Nash equilibrium and show that reflecting market behavior changes the transition pathways of individual countries substantially.

Our case study considers the transition of the electricity system of 21 countries participating in the European electricity market and demonstrates that a centralized model can underestimate costs: The annualized investment and operating costs in 2050 are 8% lower compared to the decentralized model when ignoring congestion and carbon allowance costs.

Furthermore, the installed capacity of individual countries differs substantially between centralized and decentralized models: Our results show that the installed generation capacity in a majority of countries changes by more than ±40% when market behavior is represented via bilevel optimization. The results highlight the importance of considering market behavior as it considerably affects the need for capacity expansion in individual countries.

For Germany, the largest electricity consumer in the electricity system model, the installed capacity is 49% higher in 2050 when market behavior is reflected. In addition, the technology mix differs substantially between centralized and decentralized electricity system designs for many countries.

Finally, the decentralized model identifies a system where the average market clearing prices are 40% lower than in the centralized model. The spread of country-specific locational market clearing prices is also lower in the decentralized model.

In conclusion, our study emphasizes the importance of considering market behavior in energy system models, as market behavior substantially influences stakeholders’ costs and investment strategies.

## STAR★Methods

### Key resources table

**Table:** 

REAGENT or RESOURCE	SOURCE	IDENTIFIER
**Deposited data**		

Case study data	This paper	https://doi.org/10.5281/zenodo.11124479
ecoinvent 3.7.1 (APOS)	Wernet et al.^24^	https://ecoinvent.org/

**Software and algorithms**		

Python 3.6.12	van Rossum et al.^33^	https://www.python.org/
Pyomo 5.7.3	Hart et al.^34^	http://www.pyomo.org/
Gurobi 9.0.0	Gurobi Optimization LLC^35^	https://www.gurobi.com/
SecMOD	This paper	https://doi.org/10.5281/zenodo.11124479

### Resource availability

#### Lead contact

Further information and requests for resources should be directed to and will be fulfilled by the lead contact, André Bardow (abardow@ethz.ch).

#### Materials availability

This study did not generate any new reagents.

#### Data and code availability

• The data used in our case study has been deposited at zenodo and is publicly available as of the date of publication. The DOI is listed in the key resources table. The life cycle inventory data from *ecoinvent* used in our case study cannot be deposited in a public repository because it requires a separate license.
• All original code has been deposited at zenodo and is publicly available as of the date of publication. The DOI is listed in the key resources table.
• Any additional information required to reanalyze the data reported in this paper is available from the lead contact upon request.

### Method details

#### Model description

In the following, we outline the formulation of 1) the centralized electricity system model that considers a joint objective function for all players in a single level and 2) the decentralized electricity system model that considers capacity expansion and markets in a bilevel optimization. Furthermore, to determine the Nash-equilibrium between countries for the decentralized model, we describe the tailored solution algorithms applied.

Both the centralized and the decentralized problems are formulated in the energy system modeling and optimization framework SecMOD^36^ and aim to identify a transition pathway from the initial existing capacities toward carbon-neutral electricity systems at the end of the transition horizon. To identify a transition pathway, the capacity expansion for electricity generation technologies (Further information on the case study) of all countries is determined in multiple investment years along the transition horizon following a rolling-horizon approach.^25^ The rolling horizon approach is adapted to acknowledge myopic foresight and conveniently decomposes the multi-period investment problem into computationally tractable subproblems. The capacity expansion is determined by minimizing an objective function while satisfying spatially and temporally resolved electricity demands, considering the availability and cost of electricity generation technologies, and considering additional constraints describing technical and environmental limitations.

As decision variables of the optimization problem, we consider the capacity expansion of electricity generation technologies, their operation, and the electricity transmission between countries. In the centralized approach, all decision variables are determined jointly. In the decentralized approach, the capacity expansion is determined for each country on the upper level of the bilevel problem. The operation of electricity generation technologies and electricity transmission is determined on the lower level.

#### Comparison of the centralized and decentralized modeling approaches

**Table:** 

model aspect	centralized	decentralized
strategic behavior of player	perfect cooperation	competition
upper-level objective	joint objective: total annualized cost of all players	individual objective: total annualized cost of player, including carbon-allowance & congestion
lower-level objective	–	operating cost of all players
upper-level decision variables	capacity expansion, operation of capacities, transmission	capacity expansion
lower-level decision variables	–	operation of capacities, transmission
carbon-allowance price	neglected or exogenous	formed in lower-level problem
electricity price	neglected	formed in lower-level problem
congestion management	physical limitations	physical limitations & economic considerations

The centralized and decentralized models differ in the choice and representation of the objective function: The centralized model assumes a central planner and perfect cooperation among the countries. Therefore, a joint objective function is chosen, minimizing the total annualized cost of all countries. The centralized model neglects the individual objectives of countries within an electricity system. The GHG emission limits are represented by a global constraint, eliminating the need for a carbon tax or allowances. The centralized model simplifies the problem formulation by neglecting individual objectives and trading on electricity and carbon markets, thereby eliminating opportunities for non-cooperative behavior.

In real-world electricity systems, multiple stakeholders determine the capacity expansion and follow individual, possibly conflicting interests. In addition, the electricity trade is managed on electricity markets, and GHG emissions limits are enforced by a carbon price set by either a tax or a carbon-allowance market. We model non-cooperative behavior in the decentralized approach by assuming a game situation in which the countries aim to minimize their individual costs. Considering individual countries, we can no longer limit GHG emissiosn globally on the upper level. Instead, the decentralized model includes the global GHG emission limit on the lower level of the bilevel problem, representing a carbon-allowance market that results in carbon prices (Equations 31, 32, and 33). Similarly, the decentralized model includes energy balances on the lower level of the bilevel problem, representing electricity markets that result in electricity prices (Equation 23). To minimize their individual costs, the countries invest in energy conversion capacities to generate and trade electricity and satisfy their time-dependent electricity demands.

#### Game-theoretical approach

The transition pathway of the multi-national electricity system consists of consecutive investment decisions of the players within the transition horizon. We represent the non-cooperative behavior of the players in each investment decision as a multi-leader-single-follower Stackelberg game.^6^ In the decision process, each country n∈N is a leader that minimizes its total cost by determining its capacity expansion strategy $x_{n}$. After the countries determine the electricity generation capacity expansion as leaders, the operation of the capacity $y_{n}$ is determined on the joint electricity market as the single follower. We assume that players can only influence their own strategy. For each country, we hence assume complete knowledge of 1) the other countries’ optimal capacity expansion strategies and 2) the behavior of the shared electricity and carbon markets.

For the non-cooperative game, a Nash-equilibrium^37^ is sought such that no country improve their situation by changing their own strategy (as applied in ^4^^,^^8^^,^^15^^,^^16^^,^^9^):

$$\left(\tilde{x},\tilde{y}\right)=\left(\left({\tilde{x}}_{1},{\tilde{y}}_{1}\right),\left({\tilde{x}}_{2},{\tilde{y}}_{2}\right),\ldots \left({\tilde{x}}_{\left|N\right|},{\tilde{y}}_{\left|N\right|}\right)\right)$$

Hence, the capacity expansion and operational strategy of each country n∈N is at an optimal solution from the set of optimal solutions $S_{n}\left({\left({\tilde{x}}_{\tilde{n}},{\tilde{y}}_{\tilde{n}}\right)}_{\tilde{n}\in N\setminus n}\right)$ given the strategies of all other countries ${\left({\tilde{x}}_{\tilde{n}},{\tilde{y}}_{\tilde{n}}\right)}_{\tilde{n}\in N\setminus n}$:

$${\tilde{x}}_{n},{\tilde{y}}_{n}\in S_{n}({\left({\tilde{x}}_{\tilde{n}},{\tilde{y}}_{\tilde{n}}\right)}_{\tilde{n}\in N\setminus n}),\;\forall \;n\in N$$

The total cost of each country consists of operating costs, investment costs, congestion costs on the electricity market, and carbon-allowance costs. Hence, the countries act as leaders by setting the conditions for trading on the common markets. The common markets minimize the total operating costs for supplying all electricity demands based on the available electricity generation capacities of the leaders. Hence, the markets act as a follower. The markets consist of an electricity market governing the electricity trade between countries and a carbon market constraining the total GHG emissions. The prices for electricity congestion and carbon-allowances are determined by the market model and considered by the leaders in their capacity expansion strategies.

#### Solution method

In each investment year a∈A of the transition horizon, we solve the multi-leader-single-follower Stackelberg game by dividing it into individual single-leader-single-follower Stackelberg games for each country. We iterate between the individual games via a Gauss-Seidel algorithm^38^ to obtain a Nash-equilibrium, which is a common approach in the literature (e.g., ^4^^,^^14^^,^^39^^,^^40^) due to computational efficiency for larger problem sizes compared to the solution of a complementarity problem.^40^^,^^41^ However, efficient formulations via (mixed) complementarity problems are promising, as related large-scale market equilibrium problems have been solved following this approach.^42^

In the single-leader-single-follower Stackelberg game, we assume the optimal capacity expansion strategies of all other countries to be fixed. In each single-leader-single-follower Stackelberg game, the countries have full knowledge of the behavior of the markets. Hence, we formulate the Stackelberg game of each country $\bar{n}\in N$ as a nested optimization problem (Equations 1, 2, 3, 4, 5, and 6), with the country’s objectives on the upper level (Equation 1) and the market’s objective on the lower level (Equation 3). The formulation is discussed in full detail in method details - detailed problem formulation.

(Equation 1) $$\min_{x_{\bar{n}},y}\;{C_{\bar{n}}^{\text{tot}}=C}_{\bar{n}}^{\text{inv}}\left(x_{\bar{n}}\right)+C_{\bar{n}}^{\text{op}}\left(y\right)+C_{\bar{n}}^{\text{trade}}\left(y,c_{n,e,t}^{*}\right)+C_{\bar{n}}^{CO_{2}}\left(y,c^{CO_{2}}\right)$$

(Equation 2) $$s.t.f_{\bar{n}}\left(x_{\bar{n}}\right)\leq 0$$

(Equation 3) $$y\in \arg\min_{y^{\prime }}\sum_{n\in N}C_{n}^{\text{op}}\left(y^{\prime }\right)$$

(Equation 4) $$s.t.q_{n,t}^{\text{dem}}+q_{n,t}^{\exp}\;-\;q_{n,t}^{\text{prod}}\;-\;q_{n,t}^{\text{imp}}\;-\;q_{n,t}^{\text{nsd}}=0\;:\;(c_{n,e,t}^{*}),\forall \;n\in N,t\in T$$

(Equation 5) $$\sum_{n\in N}E_{n}\leq E^{\max\;}+E^{\text{OS}}:(c^{CO_{2}})$$

(Equation 6) $$g\left(y^{\prime },x_{n}\right)\leq 0$$

The objective function in the upper level (Equation 1) includes the investment cost $C_{\bar{n}}^{\text{inv}}$, operating cost $C_{\bar{n}}^{\text{op}}$, congestion cost from trading $C_{\bar{n}}^{\text{trade}}$, and the emission cost $C_{\bar{n}}^{CO_{2}}$ of the considered country $\bar{n}$. The investment cost and operating costs in the upper-level objective depend on the upper-level variables $x_{\bar{n}}$, comprising investment decisions, and on the lower-level variables y, including the operational decisions of the electricity generation units of the considered country $\bar{n}$. In addition, the objective includes congestion and emission costs that depend on the electricity price $c_{n,e,t}^{*}$ and the carbon-allowance price $c^{CO_{2}}$ that are dual variables of the lower level. In that sense, the nested optimization problem (1)-(6) is not a conventional bilevel optimization problem. As the lower-level problem is linear and we replace it using strong duality to a single-level optimization, the presence of the dual variables in the problem formulation does not add major complications. The formulation of the cost contributors is described in detail in Equations 12, 15, 16, and 18.

The constraints in the upper level (Equation 2) govern the capacity expansion limits for the considered country $\bar{n}$.

The electricity market minimizes social welfare costs (Equation 3) and is represented in the lower level. Hence, the joint operating costs for satisfying the inelastic electricity demands are minimized by optimizing the operation of power plants of all countries n∈N, assuming known investment strategies and plant availabilities. Trading on the electricity market needs to satisfy the electricity demands. Hence, an electricity balance equation is included (Equation 4), where the electricity demand $q_{n,t}^{\text{dem}}$ and the electricity exported to neighboring countries $q_{n,t}^{\exp}$ need to equal the sum of produced electricity $q_{n,t}^{\text{prod}}$, electricity imported from neighboring countries $q_{n,t}^{\text{imp}}$, and non-served or curtailed electricity demand $q_{n,t}^{\text{nsd}}$ for all countries n∈N in all considered time steps t∈T. We assume zonal pricing of electricity with one bidding zone per decision maker. The dual variable corresponding to the electricity balance is the locational market clearing price of electricity $c_{n,e,t}^{*}$ and is considered in the congestion cost of the leader’s objective function. The curtailed electricity demand is a slack variable penalized heavily in the objective of the electricity market.

As emission trading enforces increasingly strict GHG emission limits, the GHG emissions of all countries are limited to a maximum value $E^{\max\;}$ (Equation 5). The slack variable $E^{\text{OS}\;}$ is heavily penalized in the operating costs of both upper and lower-level objectives. The dual variable corresponding to the GHG emission constraint is the carbon-allowance price $c^{CO_{2}}$ and is also considered on the upper level when determining the carbon-allowance cost of the leader. Additional lower-level constraints (Equation 6) model technical aspects of the electricity system, including operating limits of electricity generation units and the DC-load-flow model for transmission.^43^

We reformulate the bilevel problem for each country into a single-level problem via strong duality, resulting in a mathematical problem with equilibrium constraints (MPEC). The MPEC contains bilinear terms in the strong duality equation of the lower-level problem, as is standard in reformulating bilevel problems. It also contains bilinear terms in the objective function due to the presence of dual variables of the lower-level problem. As the MPEC is based on strong duality of the lower-level problem, the Karush-Kuhn-Tucker conditions of the lower-level problem are met in the feasible points of the MPEC, and thus also in the optimal solution. We, therefore, can use the stationarity and complementarity conditions of the Karush-Kuhn-Tucker conditions of the lower-level problem to reformulate the bilinear terms in the objective function of the MPEC. This substantially reduces the number of bilinear terms.^44^ The remaining bilinear term in the formulation is approximated via binary expansion.^45^ Overall, the reformulation and approximation results in a mixed-integer linear program (MILP) that can be solved using commercial solvers. Details of the reformulation of the MPEC can be found in detailed problem formulation.

The capacity expansion strategy of a country can be determined by solving the reformulated single-level problem assuming fixed strategies for all other countries. To determine the Nash-equilibrium of the capacity expansion strategies, the single-level problem is solved repeatedly in the Gauss-Seidel algorithm. We thus allow each country to adjust its investment strategy based on the investment strategies of the other countries:

**Figure:**
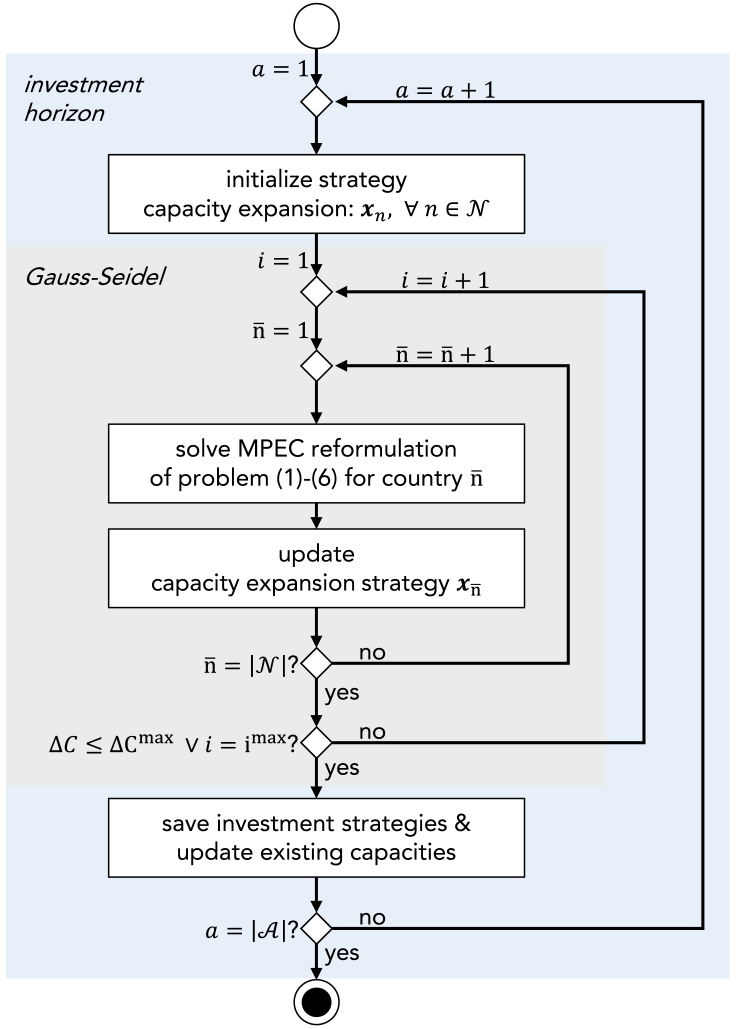
Solution method for the multi-leader-single-follower Stackelberg game The multi-leader-single-follower Stackelberg game is solved in every investment year of the investment horizon a∈A. The multi-leader-single-follower Stackelberg game is divided into single-leader-single-follower problems for each country $\bar{n}\in N$ that are reformulated to a single-level mathematical problem with equilibrium constraints (MPEC) Equations 1, 2, 3, 4, 5, and 6. The Gauss–Seidel algorithm is used to iteratively determine a Nash-equilibrium to the multi-leader-single-follower problem. The Gauss–Seidel algorithm terminates when reaching the maximum number of iterations $i=i^{\max}$ or the convergence criterion $\Delta C\leq \Delta C^{\max}$.

We initialize the Gauss-Seidel algorithm with the investment strategies of all countries from the centralized optimization. Next, we sequentially solve the MPEC for each country, assuming fixed investment decisions of all other countries. We iterate through all countries and update the capacity expansion strategy $x_{\bar{n}}$ of the considered country $\bar{n}\in N$ after solving the MPEC. The capacity expansion strategy is updated with a damping factor. In this study, a dampening factor of 0.65 yields good convergence to the Nash-equilibrium. Furthermore, we observe no substantial impact of the order of countries in the Gauss-Seidel algorithm on the final solution.

The Gauss-Seidel algorithm terminates once the maximum number of iterations $i^{\max}$ is reached, or once the relative change of the upper-level objective functions stays below a specified threshold $\Delta C={\left\|\frac{C_{n}^{\text{tot}}-C_{n-1}^{\text{tot}}}{C_{n-1}^{\text{tot}}}\right\|}_{2}\leq \Delta C^{\max}$. In this study, we set the relative convergence threshold to $\Delta C^{\max}=0.02$.

#### Optimization settings

We formulate both the centralized and the decentralized models using our energy system modeling and optimization framework SecMOD.^36^ The SecMOD framework is implemented in Python^33^ 3.6.12 and builds on the optimization package Pyomo^34^ 5.7.3. For both the centralized and decentralized models, we determine the capacity expansion of all countries in a rolling-horizon optimization without foresight. In the rolling-horizon optimization, investment decisions are made in five-year steps from 2015 to 2050, in the investment years a∈A. A maximal annualization horizon of 30 years and an interest rate of 5% are chosen. Hierarchical clustering^46^ is used to reduce the time series representing a full year with an hourly resolution to 10 typical time steps without temporal coupling.

For the comparison of centralized and decentralized model, we solve all optimization problems using Gurobi^35^ 9.0.0 on the High-Performance Cluster of RWTH Aachen University and run sequentially on one node of the Intel Xeon Platinum 8160 “SkyLake” processor with 2.1 GHz and 192 GB memory. The size of the optimization subproblems in the Gauss-Seidel algorithm depends on the parametrization specific to each country and investment period. On average, the optimization problems contain 12932 constraints, 63433 continuous variables, and 60 binary variables. The presolve-heuristics of Gurobi reduce the problem size to an average of 5757 constraints, 14383 continuous variables, and 49 integer variables, of which 42 remain binary. The total instantiation and solution times of all optimization problems is 82300 s.

The parameters settings of the Gauss-Seidel algorithm and of the Gurobi 9.0.0 solver are summarized in the table below. Note that the relative gap of 10^−4^ is the Gurobi default optimality gap and tighter than needed: We solve the MPEC to very high accuracy compared to the Gauss-Seidel algorithm.

**Table:** Optimization parameter settings for each mathematical problem with equilibrium constraints

Parameter	Value
relative gap	10^−4^
time limit	5000 s
binary expansion, number of discrete values	32
binary expansion big-M safety factor	5
Gauss-Seidel algorithm	
relative convergence threshold ΔC^max^	0.02
maximum iterations i^max^	100
damping factor	0.65

#### Further information on the case study

Our case study includes 21 countries connected by a transmission grid, modeled using the DC-load-flow approach.^43^

In total, 10 electricity production technologies are included that can be used in all countries. As conventional electricity generation technologies, we include hard coal, lignite, natural gas, and nuclear power plants. As renewable electricity generation technologies, we include onshore and offshore wind, photovoltaics, concentrated solar power, run-of-river hydropower, and geothermal power plants. We further model the existing capacities in 2015 and introduce country-specific capacity expansion limits.

**Table:** List of countries considered as stakeholder in the case study

Abbreviation	Country	Abbreviation	Country
AT	Austria	IE	Ireland
BE	Belgium	IT	Italy
BG	Bulgaria	LT	Lithuania
DE	Germany	LU	Luxembourg
DK	Denmark	LV	Latvia
EE	Estonia	NL	Netherlands
ES	Spain	PL	Poland
FI	Finland	PT	Portugal
FR	France	SE	Sweden
GR	Greece	SI	Slovenia
HR	Croatia

**Table:** Data sources for the European electricity system model

Model component	Database	Reference
existing capacities	JRC-IDEES 2015	Mantzos et al.^47^
investment & operating costs, fuel prices, and construction years of existing electricity generation capacities; lifetimes and capacity factors of conventional technologies	POTEnCIA (JRC)	European Commission,^48^, Mantzos et al.^49^
maximum capacities	ENSPRESO (JRC)	Ruiz et al.^50^
capacity factors of volatile renewable electricity generation technologies	EMHIRES (JRC)	Gonzalez Aparicio et al.^27^
Efficiencies of conventional electricity generation technologies	Eurostat	Eurostat^51^
net-transfer capacities for cross-border transmission	ENTSO-E	ENTSO-E^52^
electricity demands	ENTSO-E	ENTSO-E^53^

The European electricity system model is based on data provided by the European Union. The reference year of all data sources is 2015, with the exception of the electricity demand by ENTSO-E, which is given for 2017. Figure 1 shows the overall infrastructure for electricity production in the reference year 2015.

We aggregated transmission capacities between countries based on the reference grid of the 10-year network development plan by ENTSO-E^52^ for the reference year 2015. Furthermore, the expansion of the grid is modeled exogenously using the net transfer capacities reported in the *Global Ambition* scenario.^52^ The net transfer capacities reported by ENTSO-E are converted to a number of circuits in SecMOD, assuming a length of 25 km and a voltage level of 220 kV for each grid element due to the lack of data. Furthermore, to consider the transit through Switzerland, transmission lines are added between Germany and Italy, and between France and Austria. We neglect transmission capacities to countries outside the 21 countries considered in our study.

The hourly electricity demand is based on the electricity load data published by ENTSO-E.^53^ We choose the demands of 2017 as data for the base year 2015 is unavailable. We scaled the demand data of 2017 to arrive at the total electricity demand for 2015 of 2274 TWh/a given in the POTEnCIA model^48^ to increase data consistency.

**Table:** Lifetime and capacity factors of electricity generation technologies

Electricity generation technology	Lifetime in years	Capacity factor
coal	40	0.92
lignite	42	0.9
natural gas	32	0.93
nuclear	50	0.92
run-of-river hydro	50	0.93
geothermal	30	0.95
wind onshore	25	temporally and spatially resolved
wind onshore offshore	25	temporally and spatially resolved
photovoltaics	25	temporally and spatially resolved
concentrated solar power	30	temporally and spatially resolved

Capacity factors describe the share of an installed generation capacity that can be used, reflecting downtime due to maintenance or limited availability of intermittent renewable technologies. The capacity factors and the lifetime of the modeled electricity generation technologies are adapted from the POTEnCIA energy model.^48^ Variable capacity factors for volatile renewable electricity generation technologies are taken from the EMHIRES^27^ database. For countries where full-load hours are unavailable for wind power, we assumed similar capacity factors as in Germany as a representative country in central Europe, for which we assume a high data quality due to large wind power capacities.

We define a country-specific capacity expansion limit for each electricity generation technology. The maximum capacity of conventional power plants is assumed to be unlimited with the exception of nuclear power. For nuclear power, the maximum capacity is set depending on the national policies at the time of writing. The existing maximum capacity is unlimited in Poland, while the existing capacities can be replaced but not expanded after decommissioning in France, Slovenia, and Sweden. As the other countries have committed to phase-out nuclear power, the maximum capacity is set to zero for all other countries. The maximum capacity of geothermal and run-of-river hydro plants is assumed to have already been reached in 2015. The maximum capacity for volatile renewable electricity generation technologies is adapted from the ENSPRESO database, using 1) the central scenario for solar power plants (power density of 170 W/m^2^ and 3% land use of the available non-artificial areas) and 2) the reference scenario for onshore and offshore wind power plants (large turbines for a capacity factor exceeding 20% and a water depth of 0–60 m).

The conversion efficiencies of the electricity generation technologies for each country are based on inputs and outputs from Eurostat,^54^ leading to an efficiency averaged over the entire fleet of existing and new infrastructure. In case of data gaps, we used the country-independent efficiencies from the POTEnCIA energy model.^48^ Further, we adopt the estimates of fixed and variable operating costs and investment costs of each electricity generation technology from the POTEnCIA model.

We apply life cycle assessment to account for environmental impacts, distinguishing between infrastructure-related impacts due to the construction and disposal of processes and operational impacts. In the optimization problem, we constrain the operational impacts in line with the European target to achieve a carbon-neutral electricity system by 2050.^55^ The emission limit for 2015 is determined based on the IDEES 2015 database^47^ and is set to the total emissions of all countries included in this study in the same year. The total emission limits fall linearly to 0 by 2050 in both centralized and decentralized models. However, the European electricity system model is unable to comply with the emission limit in 2050, as even renewable electricity generation technologies are associated with small life cycle operational GHG emissions, and carbon dioxide removal technologies are excluded from our model. In 2050, life cycle operational GHG emissions are reduced by 93% and 92% compared to 2015 in the decentralized and centralized models, respectively, with residual emissions of the centralized model exceeding the decentralized model by less than 10 Mt.

The life cycle inventories (LCIs) are adapted from Baumgärtner et al.^25^ The life cycle inventory of concentrated solar power plants is modeled as in ecoinvent 3.7.1. For this study, we adapted the LCIs to account for double counting, as discussed in detail by Reinert et al.^56^ Furthermore, the LCIs of the electricity generation technologies are adapted to the location where the technologies are installed by selecting the country-specific process if possible. If a country-specific LCI is unavailable, we employ a proxy for the location using the following order: Europe without Switzerland, rest of Europe (RER), ENTSO-E, western Europe (WEU), any country from the case study, global (GLO), rest of the world (RoW).

#### Detailed problem formulation

In the following, we provide the complete bilevel problem formulation of the capacity expansion problem for the current investment year $a=\bar{a}$. For the sake of brevity, we do not include the index of the current investment year in the problem formulation.

The indices and sets are.

(1) n∈N: players, here corresponding to countries, each represented by a single node
(2) p∈P: energy converter technology
(3) a∈A: investment years including past years and current year
(4) t∈T: time step
(5) b∈B: energy carrier type
(6) l∈L: transmission line

The **upper level (UL) primal variables** $x_{n}$ for a single country $n=\bar{n}$ are:

(Equation 7) $$x_{\bar{n}}={\left\{K_{\bar{n},p}^{\text{new}}\right\}}_{p\in P}$$

(1) $K_{\bar{n},p}^{\text{new}}$ purchased capacity in the current investment year

The lower level (LL) primal variables y are:

(Equation 8) $$y={\left\{UC_{n,p,a,t},q_{n,b,t}^{\text{nsd}},q_{l,t}^{\text{tr}},\theta_{n,t},E_{n},E^{\text{OS}},C_{n}^{\text{op}}\right\}}_{n\in N,p\in P,a\in A,t\in T,b\in B,l\in L}$$

(1) $UC_{n,p,a,t}$ unit commitment
(2) $q_{n,b,t}^{\text{nsd}}$ non-served demand
(3) $q_{l,t}^{\text{tr}}$ transmitted power flow
(4) $\theta_{n,t}$ voltage level
(5) $E_{n}$ national GHG emissions
(6) $E^{\text{OS}\;}$ GHG emission overshoot
(7) $C_{n}^{\text{op}}$ national operational cost

The LL dual variables λ are:

(Equation 9) $$\lambda ={\left\{c_{n,b,t}^{*},c^{CO_{2}},{\underline{\lambda }}_{n,b,t}^{\text{nsd}},{\underline{\lambda }}_{n,p,a,t}^{\text{UC}},{\bar{\lambda }}_{n,p,a,t}^{\text{UC}},{\underline{\lambda }}_{l,t}^{\text{tr}},{\bar{\lambda }}_{l,t}^{\text{tr}},\lambda_{l,t}^{\theta },{\underline{\lambda }}^{\text{OS}},\lambda_{n}^{E},\lambda_{n}^{C}\right\}}_{n\in N,b\in B,p\in P,t\in T,a\in A,l\in L}$$

(1) $c_{n,e,t}^{*}$ locational market clearing price
(2) $c_{n,b\in B\setminus \{e\},t}^{*}$ import price
(3) $c^{CO_{2}}$ carbon allowance price
(4) ${\underline{\lambda }}_{n,b,t}^{\text{nsd}}$ dual variable corresponding to the lower limit of $q_{n,b,t}^{\mathrm{nsd}}$
(5) ${\underline{\lambda }}_{n,p,a,t}^{\text{UC}}$ dual variable corresponding to the lower limit of $UC_{n,p,a,t}$
(6) ${\bar{\lambda }}_{n,p,a,t}^{\text{UC}}$ dual variable corresponding to the upper limit of $UC_{n,p,a,t}$
(7) ${\underline{\lambda }}_{l,t}^{\text{tr}}$ dual variable corresponding to the lower limit of $q_{l,t}^{\mathrm{tr}}$
(8) ${\bar{\lambda }}_{l,t}^{\text{tr}}$ dual variable corresponding to the upper limit of $q_{l,t}^{\mathrm{tr}}$
(9) $\lambda_{l,t}^{\theta }$ dual variable corresponding to to Equation 30
(10) ${\underline{\lambda }}^{\text{OS}}$ dual variable corresponding to $E^{\mathrm{OS}}$
(11) $\lambda_{n}^{E}$ dual variable corresponding to to Equation 33
(12) $\lambda_{n}^{C}$ dual variable corresponding to Equation 15

The nested problem formulation of the capacity expansion problem of country $n=\bar{n}$ is:

(Equation 10) $$\min_{x_{\bar{n}},y}\;C_{\bar{n}}^{\text{tot}}=C_{\bar{n}}^{\text{inv}}+C_{\bar{n}}^{\text{op}}+C_{\bar{n}}^{\text{trade}}+C_{\bar{n}}^{{\text{CO}}_{2}}$$

 s.t. Equations 12, (Equation 15), (Equation 16), (Equation 17), (Equation 18), 16, 17, 18, 20, 21, and (Equation 20), (Equation 21), (Equation 22)

(Equation 11) $$y\in \arg\min_{y^{\prime }}\sum_{n\in N}C_{n}^{\text{op}}+C^{\text{OS}}$$

 s.t. Equations 23, 24, 25, 26, 27, 28, 29, 30, 31, 32, and (Equation 23), (Equation 24), (Equation 25), (Equation 26), (Equation 27), (Equation 28), (Equation 29), (Equation 30), (Equation 31), (Equation 32), (Equation 33)

The UL objective (Equation 10) is the total costs $C_{\bar{n}}^{\text{tot}}$ of country $n=\bar{n}$ and consists of the four elements:

(1) annualized investment costs $C_{\bar{n}}^{\text{inv}}$,
(2) operating costs $C_{\bar{n}}^{\text{op}}$,
(3) congestion costs resulting from trading $C_{\bar{n}}^{\text{trade}}$,
(4) carbon costs $C_{\bar{n}}^{CO_{2}}$.

The LL objective (Equation 11) represents the clearing of the electricity market and consists of two elements.

(1) the sum of operating costs $C_{n}^{\text{op}}$ over all countries n∈N, and
(2) the overshoot penalty for violating the emission constraint $C^{\text{OS}}$ if applicable.

The elements of the UL and LL objective can be expressed in terms of the primal variables and the lower level dual variables ((7)-(9)) as follows:

The annualized investment costs $C_{\bar{n}}^{\text{inv}}$ depends on the new capacities $K_{\bar{n},p}^{\text{new}}$ build in the regarded investment year $\bar{a}$ and the cost of the existing capacities $C_{\bar{n}}^{\text{inv},\text{ex}}$.

(Equation 12) $$C_{\bar{n}}^{\text{inv}}=\sum_{p\in P}\left(\frac{c_{p,\bar{a}}^{\text{inv}}}{PVF_{p}}K_{\bar{n},p}^{\text{new}}\right)+C_{\bar{n}}^{\text{inv},\text{ex}}$$

Here, $c_{p,\bar{a}}^{\text{inv}}$ is the specific investment costs of energy converter p in the investment year $\bar{a}$. $PVF_{p}$ is the technology-specific net present value factor, a parameter calculated with the interest rate i for the annualization horizon $h_{p}$.

(Equation 13) $$PVF_{p}=\frac{{\left(1+i\right)}^{h_{p}}-1}{{\left(1+i\right)}^{h_{p}}\;i},\;\;\;\forall \;p\in P$$

As annualization horizon $h_{p}$, we selected the smaller value between the maximum annualization horizon $h_{p}^{\max}=$ 30 years and the component lifetime $h_{p}^{\text{life}}$.

(Equation 14) $$h_{p}=\min\left\{h_{p}^{\max},h_{p}^{\text{life}}\;\right\},\;\;\;\forall \;p\in P$$

The cost of the existing capacities $C_{\bar{n}}^{\text{inv},\text{ex}}$ represents the annualized investment cost of previous investments that have not yet been written off. Since the annualized investment cost of existing capacities is a parameter, it only adds an offset to the objective function and does not impact the optimal solution. Here, we have included the annualized investment cost of existing capacities in the objective function for the sake of completeness as we do consider the cost of existing capacities in our analyses in the case study.

The operating costs $C_{n}^{\text{op}}$ include variable operating costs and costs for non-served demand.

(Equation 15) $$C_{n}^{\text{op}}=\sum_{t\in T}\Delta t_{t}\sum_{a\in A}\sum_{p\in P}c_{n,p,a,t}^{\text{prod},C}\;UC_{n,p,a,t}+\sum_{t\in T}\Delta t_{t}\sum_{b\in B}c_{b,t}^{\text{nsd}}q_{n,b,t}^{\text{nsd}},:\left(\lambda_{n}^{C}\right)\;\;\;\forall \;n\in N$$

Here, $\Delta t_{t}$ is the time slice weight, $UC_{n,p,a,t}$ is the utilized capacity of process p, and $c_{n,p,a,t}^{\text{prod},C}$ is the variable operating price of utilizing process p. The amount of non-served demand $q_{n,b,t}^{\text{nsd}}$ corresponds to energy carrier imports from outside the system boundary of the European energy system, while the price of non-served demand $c_{b,t}^{\text{nsd}}$, corresponds to import prices of energy carriers. As we do not allow imports of electricity from outside the system boundary of the European energy system, the non-served demand for electricity should always be zero. However, we maintain the non-served demand for electricity as a slack variable that is penalized with a prohibitively high cost of 3000 EUR/MWh.

The congestion costs $C_{n}^{\text{trade}}$ resulting from electricity trading within the system boundary is calculated using the locational market clearing price (LMCP) $c_{n,e,t}^{*}$:

(Equation 16) $$C_{n}^{\text{trade}}=\sum_{t\in T}\left(q_{n,e,t}^{\text{dem}}-q_{n,e,t}^{\text{prod}}\right)\;c_{n,e,t}^{*}\;\Delta t_{t},\;\;\;\forall \;n\in N$$

The index e indicates *electricity* and is an element of the set of energy carrier types b∈B. The exogenous electricity demand $q_{n,e,t}^{\text{dem}}$ for every country n is a parameter. The non-served electricity demand $q_{n,e,t}^{\text{nsd}}$ serves as a slack variable in the electricity balance is excluded from the congestion cost to avoid benefits of curtailing load. In the optimal solutions of our case study, the value of the slack variable is always zero. The electricity produced by each country $q_{n,e,}^{\text{prod}}$ is determined by taking the sum of electricity generated by all converter technologies p built in all investment years a:

(Equation 17) $$q_{n,e,t}^{\text{prod}}=\sum_{a\in A}\sum_{p\in P}TM_{n,p,e,a,t}UC_{n,p,a,t},\;\;\;\forall \;n\in N,t\in T$$

The conversion factors of the technology matrix $TM_{n,p,e,a,t}$ link the utilized capacity $UC_{n,p,a,t}$ of a energy converter technology p to the energy carrier b that the technology consumes or produces, where b=e corresponds to the energy carrier electricity.

The carbon cost $C_{n}^{{\text{CO}}_{2}}$ of each country n consists of the carbon allowance cost and a share of the overshoot penalty for violating the emission limit.

(Equation 18) $$C_{n}^{{\text{CO}}_{2}}=E_{n}\;c^{{\text{CO}}_{2}}+r_{n}^{\text{dem}}C^{\text{OS}},\;\;\;\forall \;n\in N\text{,}$$

with the operational GHG emissions $E_{n}$ of country n, the carbon allowance price $c^{CO_{2}}$, the total penalty for exceeding the overall emission limit $C^{\text{OS}}$, and a parameter $r_{n}^{\text{dem}}$ for allocating the overshoot penalty to the countries. The carbon allowance price $c^{CO_{2}}$ is the dual variable of the emission limit constraint in Equation 31. The overshoot penalty is allocated to the countries based on the country’s fraction of overall electricity demand:

(Equation 19) $$r_{n}^{\text{dem}}=\frac{\sum_{t\in T}\Delta t_{t}q_{n,e,t}^{\text{dem}}}{\sum_{n^{'}\in N}\sum_{t\in T}\Delta t_{t}q_{n^{'},e,t}^{\text{dem}}\;},\;\;\;\forall \;n\in N$$

The total overshoot penalty $C^{\text{OS}}$ is:

(Equation 20) $$C^{\text{OS}}=c^{\text{OS}}E^{\text{OS}}\text{,}$$

where $c^{\text{OS}}$ is the specific overshoot penalty and $E^{\text{OS}\;}$ the violation of the emission constraint. The constraints describing the violation of the emission constraint is described in Equations 31 and 32.

In addition to the objective function, UL constraints are introduced: The new capacity that can be built $K_{n,p}^{\text{new}}$ is constrained by the maximum capacity $K_{n,p}^{\max}$ and the already existing capacity $K_{n,p,a}^{\text{ex}}$.

(Equation 21) $$0\leq K_{\bar{n},p}^{\text{new}},\;\;\;\forall \;p\in P$$

(Equation 22) $$K_{\bar{n},p}^{\text{new}}\leq \max\left\{K_{\bar{n},p}^{\max}-\sum_{a\in A}K_{\bar{n},p,a}^{\text{ex}},0\right\},\;\;\;\forall \;p\in P$$

The existing capacity $K_{\bar{n},p}^{\text{ex}}$ is a parameter and indicates the capacity of converter technology p that has been built in a construction year prior to the current investment year $a<\bar{a}$ and has not yet reached the end of life. Furthermore, we apply the existing capacity $K_{\bar{n},p}^{\text{ex}}$ to model investments that have to be made in the current investment year $a=\bar{a}$.

In addition, the following LL constraints are introduced: For each country n and time step t, the power balance for electricity b=e is introduced as an equality constraint:

(Equation 23) $$q_{n,e,t}^{\text{dem}}=q_{n,e,t}^{\text{prod}}+q_{n,e,t}^{\text{nsd}}+\sum_{l\in L_{n}^{\text{in}}}q_{l,t}^{\text{tr}}+\sum_{l\in L_{n}^{out}}q_{l,t}^{\text{tr}},:\left(c_{n,e,t}^{*}\right)\;\;\;\forall \;n\in N,t\in T$$

The exogenous demand $q_{n,e,t}^{\text{dem}}$ is a parameter that can be satisfied by electricity generation $q_{n,e,t}^{\text{prod}}$ (Equation 17). Alternatively, some exogenous demand can be curtailed. The amount of electricity curtailed is considered nonserved demand $q_{n,e,t}^{\text{nsd}}$. Finally, the exogenous demand can be satisfied by electricity transmission from neighboring countries $q_{l,t}^{\text{tr}}$ on line l. The sets $L_{n}^{\text{in}}$ and $L_{n}^{\text{out}}$ contain the lines defined as going in or out of node n, respectively.

The non-served demand $q_{n,b,t}^{\text{nsd}}$ is non-negative:

(Equation 24) $$q_{n,b,t}^{\text{nsd}}\geq 0,:\left({\underline{\lambda }}_{n,b,t}^{\text{nsd}}\right),\;\;\;\forall \;n\in N,b\in B,t\in T$$

Apart from electricity b=e, all energy carrier types considered in this work b∈B∖{e} are fuels required by fossil power plants and correspond to imports from outside the system boundary:

(Equation 25) $$q_{n,b,t}^{\text{nsd}}=-\sum_{p\in P}\sum_{a\in A}TM_{n,b,p,a,t}\;UC_{n,p,a,t},:\left(c_{n,b,t}^{*}\right)\;\;\;\forall \;n\in N,b\in B\setminus \left\{e\right\},t\in T$$

The utilized capacity $UC_{n,p,a,t}$ indicates for every country n how much each converter technology p with the construction year a is used in time step t. The existing capacity $K_{n,p,a}^{\text{ex}}$ from investment years prior to the current investment year $a\in A\setminus \left\{\bar{a}\right\}$ multiplied with a time-dependent capacity factor parameter ${CF}_{n,p,t}$ is an upper bound for the utilized capacity $UC_{n,p,a,t}$ (Equation 27). Both existing capacities and capacity factor are parameters.

Accordingly, the utilized capacity of the current investment year $a=\bar{a}$ is limited by the new capacity $K_{n,p}^{\text{new}}$ and any capacities that fixed to be built exogenously in the investment year $K_{n,p,\bar{a}}^{\text{ex}}$ (Equation 28). New capacity $K_{n,p}^{\text{new}}$ can only be built in the current investment year and is a decision variable.

(Equation 26) $$0\leq UC_{n,p,a,t},:\left({\underline{\lambda }}_{n,p,a,t}^{\text{UC}}\right)\;\;\;\forall \;n\in N,p\in P,a\in A,t\in T$$

(Equation 27) $$UC_{n,p,a,t}\leq CF_{n,p,t}\;K_{n,p,a}^{\text{ex}},:\left({\bar{\lambda }}_{n,p,a,t}^{\text{UC}}\right)\;\;\;\forall \;n\in N,p\in P,a\in A\setminus \left\{\bar{a}\right\},t\in T$$

(Equation 28) $$UC_{n,p,\bar{a},t}\leq CF_{n,p,t}\;{(K_{n,p}^{\text{new}}+K}_{n,p,\bar{a}}^{\text{ex}}),:\left({\underline{\lambda }}_{n,p,\bar{a},t}^{\text{UC}}\right)\;\;\;\forall \;n\in N,p\in P,t\in T$$

$K_{n,p}^{\text{new}}$ is treated as an exogenous parameter on the LL, although it is an UL variable for the current country $\bar{n}$. For all other countries $n\in N\setminus \bar{n}$, $K_{n,p}^{\text{new}}$ is not optimized on the UL and thus a parameter. The dual variables corresponding to the upper and lower limits on $UC_{n,p,a,t}$ are ${\underline{\lambda }}_{n,p,a,t}^{\text{UC}}$ denoted and ${\bar{\lambda }}_{n,p,a,t}^{\text{UC}}$.

The transmitted power $q_{l,t}^{\text{tr}}$ of all power lines l∈L is constrained by a maximum transmittable power $q_{l,t}^{\text{tr},\max}$. The power flows in both directions in a transmission line. Thus, $q_{l,t}^{\text{tr}}$ can be positive or negative:

(Equation 29) $$-q_{l,t}^{\text{tr},\max}\leq q_{l,t}^{\text{tr}}\leq q_{l,t}^{\text{tr},\max},:\left({\underline{\lambda }}_{l,t}^{\text{tr}},{\bar{\lambda }}_{l,t}^{\text{tr}}\right)\;\;\;\forall \;l\in L,t\in T$$

The maximum capacity of the transmission lines $q_{l,t}^{\text{tr},\max}$ is a property of the transmission line type, the number of circuits, the voltage level, impedance, and resistance. The electrical properties are used to calculate the susceptance $B_{l}$. The susceptance describes the relationship between the electricity flow $q_{l,t}^{\text{tr}}$ and the difference between the voltage level $\theta_{n,t}$ between the connected countries:

(Equation 30) $$q_{l,t}^{\text{tr}}=B_{l}\;\Delta \theta_{l,t}=B_{l}\left(\theta_{n_{l}^{\text{out}},t}-\theta_{n_{l}^{\text{in}},t}\right),:\left(\lambda_{l,t}^{\theta }\right)\;\;\;\forall \;l\in L,t\in T$$

One of the two countries connected by each line needs to be defined as the origin and the other as the destination. The choice of origin and destination is necessary for the formulation of the electricity transmission and can be made arbitrarily as the flow $q_{l,t}^{\text{tr}}$ can be both positive and negative. The node that is the destination or origin of line l is defined as $n_{l}^{\text{in}}$ and $n_{l}^{\text{out}}$, respectively. The linear approximation of the alternating current transmission grid as a DC load flow is described in depth by Overbye et al.^43^

Carbon trading is reduced to the auction, neglecting banking of allowances. We treat the life cycle GHG emissions excluding infrastructure emissions $E_{n}$ as the auctioned carbon allowances for each country n. The cap $E^{\max\;}$ is set as a global constraint on the annual, operational GHG emissions of all countries. We include a non-negative GHG emission overshoot $E^{\text{OS}\;}$ as a slack variable that is penalized heavily in UL and LL objective:

(Equation 31) $$\sum_{n\in N}E_{n}\leq E^{\max}+E^{\text{OS}},:\left(c^{{\text{CO}}_{2}}\right)$$

(Equation 32) $$E^{\text{OS}}\geq 0,:\left({\underline{\lambda }}^{\text{OS}}\right)$$

The dual variable of the emission constraint (Equation 31) is the carbon allowance price $c^{CO_{2}}$.

The operational GHG emissions $E_{n}$ are calculated with the specific emissions for converter technology $c_{n,p,a,t}^{\text{prod},E}$.

(Equation 33) $$E_{n}=\sum_{t\in T}\Delta t_{t}\sum_{a\in A}\sum_{p\in P}c_{n,p,a,t}^{\text{prod},E}\;UC_{n,p,a,t},:\left(\lambda_{n}^{E}\right)\;\;\;\forall \;n\in N$$

The bilevel problem (10)-(33) is reformulated to a single-level Mathematical Program with Equilibrium Constraints (MPEC) via strong duality of the LL. The strong duality equation is:

(Equation 34) $$\sum_{n\in N}C_{n}^{\text{op}}+c^{\text{OS}}E^{\text{OS}}+\sum_{t\in T}\Delta t_{t}\left(-\sum_{n\in N}q_{n,e,t}^{\text{dem}}\;c_{n,e,t}^{*}+\sum_{n\in N}\sum_{p\in P}\sum_{a\in A\setminus \bar{a}}CF_{n,p,t}K_{n,p,a}^{\text{ex}}{\bar{\lambda }}_{n,p,a,t}^{\text{UC}}+\sum_{n\in N}\sum_{p\in P}CF_{n,p,t}\left(K_{n,p}^{\text{new}}+K_{n,p,\bar{a}}^{\text{ex}}\right){\bar{\lambda }}_{n,p,\bar{a},t}^{\text{UC}}+\sum_{l\in L}q_{l,t}^{\text{tr},\max}{\underline{\lambda }}_{l,t}^{\text{tr}}+\sum_{l\in L}q_{l,t}^{\text{tr},\max}{\bar{\lambda }}_{l,t}^{\text{tr}}\right)+E^{\max}\;c^{{\text{CO}}_{2}}=0$$

The reformulation to an MPEC results in bilinear terms in the strong duality equation:

(Equation 35) $$K_{\bar{n},p}^{\text{new}}{\bar{\lambda }}_{\bar{n},p,\bar{a},t}^{\text{UC}},\;\;\;\forall \;p\in P,t\in T$$

Note that $K_{n,p}^{\text{new}}$ is a variable for the current country $n=\bar{n}$. For all other countries $n\in N\setminus \bar{n}$, $K_{n,p}^{\text{new}}$ is not optimized on the UL and thus a parameter.

Furthermore, bilinear terms occur in the objective function of the MPEC (terms (36) and (37)).

For computational performance, we aim for a mixed-integer linear programming formulation of the single-level problem. Therefore, the objective function is reformulated following the approach described by Ruiz and Conejo,^44^ to address the bilinear terms (36) and (37) in the objective function of the MPEC.

(Equation 36) $$-\sum_{t\in T}\Delta t_{t}\;c_{\bar{n},e,t}^{*}\sum_{a\in A}\sum_{p\in P}TM_{\bar{n},p,e,a,t}UC_{\bar{n},p,a,t}$$

(Equation 37) $$E_{\bar{n}}\;c^{{\text{CO}}_{2}}$$

The reformulation uses selected stationarity (Equation 32) and complementarity conditions (Equation 38) of the Karush-Kuhn-Tucker conditions of the LL problem.

In particular, the used stationarity conditions, with the Lagrangian function L are:

(Equation 38) $$\frac{\partial L}{\partial UC_{n,p,a,t}}:0=\Delta t_{t}\left(-\sum_{b\in B}TM_{n,b,p,a,t}\;c_{n,b,t}^{*}-{\underline{\lambda }}_{n,p,a,t}^{\text{UC}}+{\bar{\lambda }}_{n,p,a,t}^{\text{UC}}-c_{n,p,a,t}^{\text{prod},E}\;\lambda_{n}^{E}-c_{n,p,a,t}^{\text{prod},C}\lambda_{n}^{C}\right)\text{,}\;\;\;\forall \;n\in N,p\in P,a\in A,t\in T$$

(Equation 39) $$\frac{\partial L}{\partial q_{n,b,t}^{\text{nsd}}}:0=\Delta t_{t}\left(-\;c_{n,b,t}^{*}-{\underline{\lambda }}_{n,b,t}^{\text{nsd}}-c_{b,t}^{\text{nsd}}\lambda_{n}^{C}\right),\;\;\;\forall \;n\in N,b\in B,t\in T$$

(Equation 40) $$\frac{\partial L}{\partial E_{n}}:0=\lambda_{n}^{E}+c^{{\text{CO}}_{2}},\;\;\;\forall \;n\in N$$

(Equation 41) $$\frac{\partial L}{\partial C_{n}^{\text{op}}}:0=1+\lambda_{n}^{C},\;\;\;\forall \;n\in N$$

Furthermore, the used complementarity conditions of Equations 27, 28, and 24 are:

(Equation 42) $${\underline{\lambda }}_{n,p,a,t}^{\text{UC}}\;UC_{n,p,a,t}=0,\;\;\;\forall \;n\in N,p\in P,a\in A,t\in T$$

(Equation 43) $$\left(CF_{n,p,t}K_{n,p,a}^{\text{ex}}-UC_{n,p,a,t}\right){\bar{\lambda }}_{n,p,a,t}^{\text{UC}}=0,\;\;\;\forall \;n\in N,p\in P,a\in A\setminus \bar{a},t\in T$$

(Equation 44) $$\left(CF_{n,p,t}\left(K_{n,p}^{\text{new}}+K_{n,p,\bar{a}}^{\text{ex}}\right)-UC_{n,p,\bar{a},t}\right){\bar{\lambda }}_{n,p,\bar{a},t}^{\text{UC}}=0,\;\forall \;n\in N,p\in P,t\in T$$

(Equation 45) $$q_{n,b,t}^{\text{nsd}}{\underline{\lambda }}_{n,b,t}^{\text{nsd}}=0,\;\;\;\forall \;n\in N,b\in B,t\in T$$

The nonlinear term (36) can be reformulated using Equation 38:

(Equation 46) $$-\sum_{t\in T}\Delta t_{t}\sum_{a\in A}\sum_{p\in P}c_{\bar{n},e,t}^{*}\;TM_{\bar{n},e,p,a,t}\;UC_{\bar{n},p,a,t}=\sum_{t\in T}\Delta t_{t}\sum_{a\in A}\sum_{p\in P}\left(\sum_{b\in B\setminus e}TM_{\bar{n},b,p,a}\;c_{\bar{n},b,t}^{*}+{\underline{\lambda }}_{\bar{n},p,a,t}^{\text{UC}}+{\bar{\lambda }}_{\bar{n},p,a,t}^{\text{UC}}+c_{\bar{n},p,a,t}^{\text{prod},E}\lambda_{\bar{n}}^{E}+c_{\bar{n},p,a,t}^{\text{prod},C}\lambda_{\bar{n}}^{C}\right)UC_{\bar{n},p,a,t}$$

The reformulation of term (36) results in additional nonlinearities in Equation 46:

(Equation 47) $$\sum_{t\in T}\Delta t_{t}\sum_{a\in A}\sum_{p\in P}\sum_{b\in B\setminus e}TM_{\bar{n},b,p,a}\;c_{\bar{n},b,t}^{*}\;UC_{\bar{n},p,a,t}$$

(Equation 48) $$\sum_{t\in T}\Delta t_{t}\sum_{a\in A}\sum_{p\in P}\left({\underline{\lambda }}_{\bar{n},p,a,t}^{\text{UC}}UC_{\bar{n},p,a,t}-{\bar{\lambda }}_{\bar{n},p,a,t}^{\text{UC}}UC_{\bar{n},p,a,t}\right)$$

(Equation 49) $$\sum_{t\in T}\Delta t_{t}\sum_{a\in A}\sum_{p\in P}c_{\bar{n},p,a,t}^{\text{prod},E}\;\lambda_{\bar{n}}^{E}\;UC_{\bar{n},p,a,t}$$

(Equation 50) $$\sum_{t\in T}\Delta t_{t}\sum_{a\in A}\sum_{p\in P}c_{\bar{n},p,a,t}^{\text{prod},C}\lambda_{\bar{n}}^{C}\;UC_{\bar{n},p,a,t}$$

Nonlinear term (47) can be further reformulated to a linear expression using Equations 25, 39, 45, and 41:

(Equation 51) $$\sum_{t\in T}\Delta t_{t}\sum_{a\in A}\sum_{p\in P}\sum_{b\in B\setminus e}TM_{\bar{n},b,p,a}\;c_{\bar{n},b,t}^{*}\;UC_{\bar{n},p,a,t}=-\sum_{t\in T\;}\Delta t_{t}\sum_{b\in B\setminus e}q_{\bar{n},b,t}^{\text{nsd}}c_{b,t}^{\text{nsd}}$$

Nonlinear term (48) can be reformulated using (Equation 42), (Equation 43), (Equation 44), 43, and 44:

(Equation 52) $$\sum_{t\in T}\Delta t_{t}\sum_{a\in A}\sum_{p\in P}\left({\underline{\lambda }}_{\bar{n},p,a,t}^{\text{UC}}UC_{\bar{n},p,a,t}-{\bar{\lambda }}_{\bar{n},p,a,t}^{\text{UC}}UC_{\bar{n},p,a,t}\right)=-\sum_{t\in T}\Delta t_{t}\sum_{p\in P}\left(\sum_{a\in A\setminus \bar{a}}CF_{\bar{n},p,t}K_{\bar{n},p,a}^{\text{ex}}{\bar{\lambda }}_{\bar{n},p,a,t}^{\text{UC}}+CF_{\bar{n},p,t}\left(K_{\bar{n},p,\bar{a}}^{\text{ex}}+K_{\bar{n},p}^{\text{new}}\right){\bar{\lambda }}_{\bar{n},p,\bar{a},t}^{\text{UC}}\right)$$

Nonlinear term (49) can be reformulated using Equations 33 and 40:

(Equation 53) $$\sum_{t\in T}\Delta t_{t}\sum_{a\in A}\sum_{p\in P}c_{\bar{n},p,a,t}^{\text{prod},E}\;\lambda_{\bar{n}}^{E}\;UC_{\bar{n},p,a,t}=E_{\bar{n}}\lambda_{\bar{n}}^{E}=-E_{\bar{n}}c^{{\text{CO}}_{2}}$$

Nonlinear term (50) can be reformulated using Equation 41

(Equation 54) $$\sum_{t\in T}\Delta t_{t}\sum_{a\in A}\sum_{p\in P}c_{\bar{n},p,a,t}^{\text{prod},C}\lambda_{\bar{n}}^{C}\;UC_{\bar{n},p,a,t}=-\sum_{t\in T}\Delta t_{t}\sum_{a\in A}\sum_{p\in P}c_{\bar{n},p,a,t}^{\text{prod},C}\;UC_{\bar{n},p,a,t}$$

Inserting the reformulated terms (51)-(54) into the objective function (Equation 10) results in:

(Equation 55) $$C_{\bar{n}}^{\text{tot}}=C_{\bar{n}}^{\text{inv}}+\sum_{t\in T}\Delta t_{t}\sum_{a\in A}\sum_{p\in P}c_{\bar{n},p,a,t}^{\text{prod},C}\;UC_{\bar{n},p,a,t}+\sum_{t\in T}\Delta t_{t}\sum_{b\in B}c_{b,t}^{\text{nsd}}q_{\bar{n},b,t}^{\text{nsd}}+\sum_{t\in T}{\Delta t_{t}c}_{\bar{n},e,t}^{*}\left(q_{\bar{n},e,t}^{\text{dem}}-\sum_{a\in A}\sum_{p\in P}TM_{\bar{n},p,e,a,t}\;UC_{\bar{n},p,a,t}\right)+E_{\bar{n}}\;c^{{\text{CO}}_{2}}+r_{\bar{n}}^{\text{dem}}C^{\text{OS}}=C_{\bar{n}}^{\text{inv}}+\sum_{t\in T}{\Delta t_{t}c}_{\bar{n},e,t}^{*}q_{\bar{n},e,t}^{\text{dem}}+\sum_{t\in T}\Delta t_{t}c_{e,t}^{\text{nsd}}q_{\bar{n},e,t}^{\text{nsd}}-\sum_{t\in T}\Delta t_{t}\sum_{p\in P}CF_{\bar{n},p,t}\left(\sum_{a\in A\setminus \bar{a}}K_{\bar{n},p,a}^{\text{ex}}{\bar{\lambda }}_{\bar{n},p,a,t}^{\text{UC}}+\left(K_{\bar{n},p,\bar{a}}^{\text{ex}}+K_{\bar{n},p}^{\text{new}}\right){\bar{\lambda }}_{\bar{n},p,\bar{a},t}^{\text{UC}}\right)+r_{\bar{n}}^{\text{dem}}C^{\text{OS}}$$

The remaining nonlinear term in the reformulated objective function (Equation 55) also occurs in the strong duality (Equation 34):

(Equation 56) $$K_{\bar{n},p}^{\text{new}}\;{\bar{\lambda }}_{\bar{n},p,\bar{a},t}^{\text{UC}},\forall \;p\in P,t\in T$$

The remaining nonlinear term (56) is approximated via binary expansion^45^ to arrive at a MILP formulation. The primal variable $K_{\bar{n},p}^{\text{new}}$ is discretized using 32 discrete values.

For each investment period, the MPEC is solved for all countries using a diagonalization scheme. Before proceeding with the next investment period,

(1) we update existing capacities $K_{n,p,a}^{\text{ex}}$ by including newly built capacities, and by removing capacities that are retired, and
(2) we update annualized investment costs $C_{n}^{\text{inv},\text{ex}}$ by including the annualized cost of newly built capacities and excluding investments that are written off.
